# *Epilepsia Open* – June 2025 Announcements

**DOI:** 10.1002/epi4.70071

**Published:** 2025-06-13

## ILAE CONGRESSES

Curso virtual sobre epilepsia en atención primaria para América Latina 2025

2 June ‐ 27 July 2025

Online

Curso Online de Status Epiléptico 2025

6 June ‐ 31 July 2025

Online

Cápsula virtual sobre primera crisis para América Latina 2025

30 June ‐ 6 July 2025

Online

15th ILAE School for Neuropathology and Neuroimaging in Epilepsy

31 July ‐ 3 August 2025

Campinas, São Paulo, Brazil

XVIII Workshop on Neurobiology of Epilepsy (WONOEP 2025)

25 ‐ 29 August 2025

Portugal

36th International Epilepsy Congress

30 August ‐ 3 September 2025

Lisbon, Portugal

## 2026

15th ILAE School on Pre‐Surgical Evaluation for Epilepsy and Epilepsy Surgery

19 ‐ 23 January 2026

Brno, Czech Republic

5th International Summer School of Neuropsychology

19 ‐ 24 April 2026

Picardy, France

16th European Epilepsy Congress

5 ‐ 9 September 2026

Athens, Greece

## ILAE WEBINARS

Febrile seizures

3 June 2025

AOEC Webinar Series: Epilepsy genetics: where new discoveries could transform the future

4 June 2025

Les épilepsies myoclono‐atoniques

10 June 2025

AOEC Webinar Series: Overcoming access to epilepsy care with new technologies

12 June 2025

Understanding Functional Seizures in Children

17 June 2025

Neuromodulation: How can we optimize DBS protocols?

18 June 2025

ILAE e‐Forum: Diagnosis and treatment of refractory status epilepticus

26 June 2025

ILAE e‐Forum: The role of digital technologies in the management of epilepsy

4 November 2025

ILAE e‐Forum: EEG: When, why and how?

12 December

## OTHER CONGRESSES

### International Conference for Technology and Analysis of Seizures (ICTALS 2025)

2 ‐ 6 June 2025

Montreal, Canada

### 10th Mediterranean Neuroscience Society Conference

7 ‐ 11 June 2025

Chania, Crete, Greece

### The Comorbidities of Epilepsy Course

20 June 2025

London, UK & Online

### Korean Epilepsy Congress 2025

20 ‐ 21 June 2025

Seoul, South Korea

### 11th Congress of the European Academy of Neurology

21 ‐ 25 June 2025

Helsinki, Finland

### 11th Combined Migrating Caucasian Course on Epileptology (CMCCE)

3 ‐ 6 July 2025

Tbilisi, Georgia

### 21st San Servolo Advanced Epilepsy Course

21 July ‐ 1 August 2025

San Servolo (Venice), Italy

### XVIII Congreso de la Liga Colombiana Contra la Epilepsia

24 ‐ 27 July 2025

Cali, Colombia

### 5th International Congress on Mobile Health and Digital Technology in Epilepsy

4 ‐ 6 September 2025

Copenhagen, Denmark

### 19th European Congress for Clinical Neurophysiology

9 ‐ 12 September 2025

London, UK

### ILAE British Young Epilepsy Section (YES) Early Career Building Day

14 September 2025

Bournemouth, UK

### ILAE British Branch 2025 Annual Scientific Conference

15 ‐ 17 September 2025

Bournemouth, UK

### 9th Global Symposium on Ketogenic Therapies

17 ‐ 19 September 2025

Paris, France

### 19th Panhellenic Congress of Epilepsy

25 ‐ 28 September 2025

Heraklion, Crete, Greece

### 7th Bologna EPIPED‐EEG Course: Reflex Epilepsies: Mechanisms and Electro‐Clinical Manifestations

27 September ‐ 1 October 2025

Bologna, Italy

### 2025 CLAE Annual Scientific Meeting

24 ‐ 26 October 2025

Calgary, Alberta, Canada

### Epilepsy Society of Australia 39th Annual Scientific Meeting

5 ‐ 7 November 2025

Perth, Australia

### 42nd International Conference on Advances in Psychiatry and Mental Health

10 ‐ 11 November 2025

Dubai, UAE

### In Search of Lost Time 6

12 ‐ 14 November 2025

Rome, Italy

### The 2025 ILAE British Branch Clinical Epilepsy 1‐Day Course for Doctors in Training & Others

22 November 2025

Birmingham, UK

### Encephalitis 2025

3 ‐ 4 December 2025

London, UK & Online

## 2026

18th Eilat Conference on New Antiepileptic Drugs and Devices

3 ‐ 6 May 2026

Madrid, Spain

